# Efforts Towards Polio Eradication in Madagascar: 1997 to 2017

**DOI:** 10.29245/2578-3009/2021/S2.1102

**Published:** 2021-04-13

**Authors:** Marcellin Mengouo Nimpa, Noёline Ravelomanana Razafiarivao, Annick Robinson, Mamy Randriatsarafara Fidiniaina, Richter Razafindratsimandresy, Yolande Vuo Masembe, Christiane Ramonjisoa Bodohanta, Isidore Koffi Kouadio, Issa Kana kode Nyazy, Moussa Simpore, Charlotte Faty Ndiaye, Joseph Chukwudi Okeibunor

**Affiliations:** 1 World Health Organization Regional Office for Africa, Congo; 2 Faculté de Médécine d’Anatanarivo, Madagascar; 3 Centre Hospitalier Universitaire Mère Enfant Tsaralalana (CHUMET) -Antananarivo; 4 Direction Centrale du Service Militaire d’Antanarivo; 5 Institut Pasteur de Madagascar; 6 Expanded Programme of Immunization, Madagascar

**Keywords:** Poliomyelitis, Polio eradication, Madagascar, Epidemiology, Polio free status, Certification

## Abstract

**Background:**

In 1988, the World Health Assembly launched the Global Polio Eradication Initiative. WHO AFRO is close to achieve this goal with the last wild poliovirus detected in 2014 in Borno States in Nigeria. The certification of the WHO African Region requires that all the 47 member states meet the critical indicators for a polio free status. Madagascar started implementing polio eradication activities in 1996 and was declared polio free in June 2018 in Abuja. This study describes the progress achieved towards polio eradication activities in Madagascar from 1977-2017 and highlights the remaining challenges to be addressed.

**Methods:**

Data were collected from the national routine immunization services, Country Acute Flaccid surveillance databases and national reports of SIAS and Mop Up campaign. Country complete polio and immunization related documentation provided detailed historical information’s.

**Results:**

From 1997 to 2017, Madagascar reported one wild poliovirus (WPV) outbreak and four circulating Vaccine Derived Polio Virus (cVDPV) oubreaks with a total of 21 polioviruses (1 WPV and 21 cVDPV). The last WPV and cVDPV were notified in 1997 in Antananarivo and 2015 in Sakaraha health districts respectively. Madagascar met the main polio surveillance indicators over the last ten years and made significant progress following the last cVDPV2 outbreak in 2014 -2015. In addition, the country successfully implemented the switch from trivalent Oral Polio Vaccine (tOPV) to bivalent Oral Polio vaccine (bOPV) and containment activities. Environmental Surveillance established since 2015 did not reveal any poliovirus. The administrative coverage of the 3rd dose of oral polio vaccine (OPV3) varied across the years from 55% in 1991 to a maximum of 95% in 2007 before a progressive decrease to 86% in 2017. The percentage of AFP cases with more than 3 doses of oral polio vaccines increased from 56% in 2014 to 88% in 2017. A total of 19 supplementary immunization activities (SIA) were conducted in Madagascar from 1997 to 2017, among which 3 were subnational immunization days (sNID) and 16 were national immunization days (NIDs). Poor routine coverage contributed to the occurrence of cVDPC outbreaks in the country; addressing this should remain a key priority for the country to maintain the polio free status.

From 2015 to June 2017, Madagascar achieved the required criteria leading to the acceptance of the country’s polio-free documentation in June 2018 by ARCC. However, continuous efforts will be needed to maintain a highly sensitive polio surveillance system with emphasis on security compromised areas. Finally strengthening the health system and governance at all levels will be necessary if these achievements are to be sustained.

**Conclusions:**

High national political commitment and support of the Global Polio Eradication Partnership were critical for Madagascar to achieve polio free status. Socio-political instability, weakness of the health system, sub-optimal routine immunization performance, insufficient SIA quality and existing security compromised areas remain critical program challenges to address in order to maintaining the polio free status. Continuous high-level advocacy should be kept in order to ensure that new government authorities maintain polio eradication among the top priorities of the country.

## Introduction

Eradicating poliomyelitis is one of the most important public health priorities since the launch of the Global Polio Eradication Initiative (GPEI) in 1988. Four main polio eradication strategies were recommended and implemented worldwide. These include strengthening routine immunization services, acute flaccid paralysis (AFP) surveillance, implementing quality preventive or reactive Supplementary Immunization Activities (SIA’s) and mopping up campaigns.

Implementing the polio eradication strategies listed above has contributed 99% reduction polio cases globally in 2018 compared to 350 000 children who were paralyzed each year before the launch of GPEI. As at 2018, only three countries, namely Afghanistan, Pakistan and Nigeria, remained endemic with Wild Polio Virus (WPV); WHO estimates that more than 10 million polio paralysis have been prevented worldwide since the launch of the GPEI.

WHO African region is close to eradicate poliovirus. The last confirmed polio case occurred in 2017 in Borno State in Nigeria. The certification of the WHO African Region as polio free requires that all the 47 member states meet the critical indicators for a polio free status and have their polio certification status confirmed by the African Regional Certification Commission (ARCC).

Madagascar commenced implementation of polio eradication activities in 1996 and the ARCC declared it polio free in June 2018 following a review of country documentation and field verifications, thus becoming the 40^th^ polio free country in the African region. Achieving polio free status is a critical step in the polio eradication process and a recognition of progress achieved by the country and GPEI partners.

However, the efforts that contributed to this achievement of polio free status in Madagascar has not been documented anywhere. Such documentation helps to ensure that maximum benefit can be accrued from lessons learnt from outbreak response activities. This paper, therefore documents and describes the efforts towards polio eradication in Madagascar from 1977-2017 and highlights the remaining challenges to be addressed. It also makes recommendations for maintaining polio free status until the world is certified polio free. This work also presents lessons learned to guide future efforts in improving routine immunization services, AFP Surveillance system and SIA.

## Methodology

### Study Area and population

Madagascar is one of the biggest island country in the world. Located in the Indian Ocean, it covers 587,041 km^2^ and is separated from the African continent by the Mozambique channel. The country is divided into 22 administrative regions and 114 health districts. In 2017, the population was estimated at 25,530,500 inhabitants with children under 1 year representing 3,33% of the population and those under 15 years 45% (ref, *Ministère de la santé;Sectorisation, Déc. 2017)*

### Population/persons

#### Data collection techniques

##### Polio eradication activities / Surveillance System in the country

In order to obtain a polio free status, Madagascar implemented the four recommended main polio eradication strategies namely: routine immunization strengthening, AFP surveillance system, mop up campaigns and supplementary immunization activities (SIA).

The Ministry of Public Health (MoH) has been coordinating Polio eradication activities in the country through the Expanded Programme of Immunization (EPI) Direction which is directly attached to the General Secretary of the MoH. The EPI programme manager ensures technical implementation and monitoring of polio eradication activities with local GPEI partners whereas the administrative coordination is ensured by the Interagency Coordination Committees (IACC). Three polio committees, namely the National Certification committee (CNC), the National Polio Expert committee (CNEP) et National Technical Containment Group (NGTF) made up of independent experts from universities and private sectors have been functional providing guidance to the EPI and IACC on the implementation of polio eradication activities with emphasis on strategies to overcome the gaps identified.

### Surveillance System (Acute Flaccid Paralysis and environmental) and laboratory

Acute flaccid paralysis surveillance was introduced in 1996 in Madagascar and is implemented following WHO African Region (AFRO) and US Centers for Disease Control and Prevention (CDC) guidelines. A case of Acute Flaccid Paralysis is defined as *“Any child under 15years of age with sudden onset of paralysis (weakness of the limb – arm, leg or both)”,* or *“any person of any age in whom a clinician suspects polio”.* For each AFP case, two stool samples are collected 24 to 48 hours apart and sent to the laboratory within 72 hours after the second stool for confirmation. (WHO 2013a)

In order to effectively carry out AFP surveillance in Madagascar, the EPI programme proceeded by identifying surveillance focal points at district, regional and central levels including other health personnel and community volunteers. They were trained on AFP case identification, sample collection and transportation to the Laboratory. Surveillance sites included both health facilities and traditional healers. They were categorized into high priority, medium priority and low priority. Surveillance focal points received regular funding from WHO, which enable them to carry out active surveillance visits based on the level of priority. A set of indicators are used to monitor AFP surveillance system performance, the key ones are i) Non-polio AFP rate for 100000 children under 15 years which measures the sensibility of the surveillance system. The GPEI recommends as certification standards a non-polio AFP rate of at least 1 per 100,000 under 15 years population in non-endemic countries and at least 2/100,000 under 15 years population rate in endemic countries. Ii) The proportion of children with AFP detected within 14 days from the onset of the paralysis measures, which measures the capacity of the system to detected AFP cases early and is set at 80% as a minimum (MoH Madagascar 2013).

Environmental surveillance (ES) on the other hand consists of regular collection of sewage water and analysis at the laboratory to identify poliovirus. This activity was introduced in 2015 in Madagascar to complement AFP surveillance within the context of the Polio Endgame. Environmental surveillance complements AFP surveillance and is based on the rational that only 1-5% of poliovirus carriers manifest with paralysis. This consisted of periodic testing of sewage samples from sewage for identification of any poliovirus in well-identified sites.

Laboratory plays a critical role in AFP and environmental surveillance system in Madagascar. The national reference laboratory, Institut Pasteur de Madagascar based in the capital Antanarivo is accredited annually. AFP and environmental surveillance samples collected from the field are sent by health districts to the laboratory. Most of these samples are sent by road using public transports and few by air using sample carriers and keeping them cold during the transport. Any suspicious case of poliovirus is secondarily sent to Regional Reference Laboratory based in Johannesburg, South Africa, for sequencing, final confirmation and classification as wild poliovirus (WPV) or circulating or ambiguous vaccine derive poliovirus (VDPV).

### Routine immunization services

Routine immunization services are delivered in health facilities including frontline health facilities and hospitals. Polio vaccines are delivered with other childhood vaccines either at fixed posts in the health facility or during outreach services. The vaccination schedule for polio is one dose at birth, followed by additional doses at 6, 10, and 14 weeks of age. Administrative immunization coverage is used to monitor programme performance. This is calculated by counting the number of children who received at least 3 doses of Polio vaccines divided by the total target population of children under 1 year.

### Supplementary immunization activities and quality assurance

Supplementary immunization Activities are implemented in collaboration with community volunteers thanks to door-to-door strategy. Different types of Oral Polio vaccines (tOPV, bOPV, mOPV) have been used during SIAs. However, it is important to mention that, since the end of April 2016, tOPV has been withdraw and replaced with bOPV for SIAs and routine immunization. Since the switch, there is no longer tOPV available on the field. Independent monitoring and LQAS are used to assess the quality of SIAs in line with WHO recommendations.

### Mop Up Immunization campaigns

Mop-up campaigns are door-to-door immunizations that are carried out in specific areas where the virus is known or suspected to still be circulating. Priority areas include those where polio cases have been found over the previous three years and where access to health care is difficult. Other criteria include high population density, high population mobility, poor sanitation, and low routine immunization coverage. (WHO, Polio Strategic plan 2019-2023). As recommended by the GPEI, Madagascar implemented Mop Up campaigns in high risk areas following detection of any poliovirus either WPV or VDPV. These were organized using the door-to-door strategy. Vaccines used and the targeted population depended on the epidemiology and genetical characteristics of the virus.

### Data collection and Analysis

Data was collected from the national routine immunization services, SIAs and surveillance databases. The AFP surveillance database was analyzed using EPI Info package version 3.5.1. Standard indicators were used to assess the performance of different components of the polio eradication activities in the country.

## Key Results

### Routine Immunization Services

**Figure 1:**
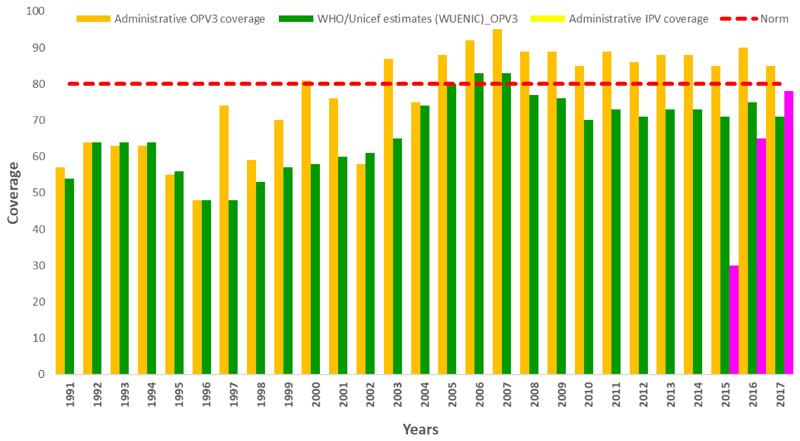
Trend in OPV3 and IPV coverage from 1991 to 2017 (Administrative and WHO-Unicef estimates)

The administrative coverage of the 3^rd^ dose of the oral polio vaccine (OPV3) varied across the years, from 55% in 1991 to a maximum of 95% in 2007 before a progressive decrease to 86% in 2017 as shown in figure 1. The three regions with the lowest administrative immunization coverage over the last ten years (2008-2017) were Melaky (78%), South-East (79.8%) and Boeni (81.1%). Ihrombe (95%), Vakinakatra (94.2%) and Analanjirofo (93,4%) registered the highest administrative coverage during the same period.

United Nations Children’s Fund (UNICEF) and World Health Organization (WHO) estimates however show discrepancies over the years with a maximum of 16 points with the administrative coverage. The national OPV3 based on UNICEF/WHO estimates varied from 50% in 1991 to a maximum of 83% in 2007 and dropped to 71% in 2017.

The percentage of AFP cases who received >=3 doses of Polio vaccines increased from 56% in 2014 to 88% in 2017.

The highest percentage of zero-dose children was recorded in the three regions of Analamanga (7%), Mélaky (14%) and Vakinankaratra (3%).

The Inactivated Polio Vaccine (IPV) vaccine was introduced in the national immunization programme in April 2015 as African countries were preparing the switch, which took place on the 25^th^ April 2016 in Madagascar. IPV administrative coverage increased from 30% in 2015 to 74% in 2017.

## AFP Surveillance I Poliovirus Surveillance and Reported Poliovirus Cases

### Acute Flaccid Paralysis Surveillance

Acute Flaccid Paralysis surveillance was introduced in Madagascar in 1996 as part of the polio eradication strategies mainly as passive surveillance. Active surveillance was integrated in 1999 following the programme review. From 2008 to 2017, a total of 4014 AFP cases were reported in Madagascar with an average of 402 AFP cases per year. The range varied from 183 in 2008 to a maximum of 788 in 2016 (Table 2).

Madagascar met the main polio surveillance indicators from 2008-2017) and made significant progress following the last cVDPV2 outbreak in 2014 -2015. The annual non-Polio AFP rate per 100,000 Under 15 years population and stool adequacy were above 1 and 80% respectively. The national non-Polio AFP increased from 2.5 in 2008 to 6.02 in 2017. From 2015 to 2017, the highest annual non-Polio AFP rate was in Haute Matsiatra (6.78), Menabe (5.94) and Betsiboka (5.31) regions while the lowest rates were in Itasy (1.42), Aloatra Mangoro (2.48) and Bongolava (2.56) regions (Table2).

The proportion of AFP cases with adequate stool specimens varied from 63% in 2015 to 93% in 2017 at the national level. During this same period, the highest AFP proportion with stool adequacy per region were observed in Bongolava (97%), Analamanga (93%) and Itasy (93%) regions and the lowest in Melaky (47.7%), South-East (60%) and Androy (67.3%) regions.

This improvement was also noted at subnational level where just 1(4.5%) out of the 22 regions met both AFP surveillance core indicators in 2015 as opposed to 21(95%) in 2017. Likewise, the proportion of districts meeting both surveillance indicators increased from 24% in 2015 to 71% in 2017.

### Environmental surveillance

Environmental Surveillance was established in 2015 within the context of responding to the 2014-15 cVDPV polio outbreak in three high risk regions, namely Analamanaga, Anosy, Sud-West and Boeny. A total of 17 sites were functional in December 2017. Samples were regularly collected with a completeness of more than 99%.

Since the establishment of the environmental surveillance, no wild poliovirus (WPV) or vaccine derived poliovirus (VDPV) has been detected. No type 2 poliovirus has been detected after the switch. Non polio enteroviruses were detected in about 45% of the samples

### WPV and cVPDV epidemiology and AFP surveillance

**Figure 3:**
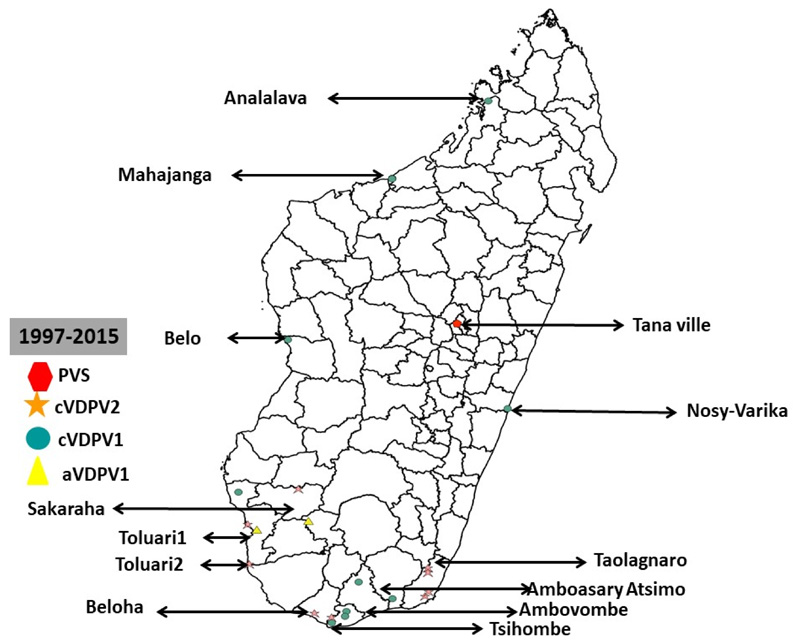
Geographic distribution of Wild poliovirus and circulating Vaccine Derved Polio Virus from 1997 to 2017, in Madagascar

From 1997 to 2017, Madagascar reported one WPV outbreak and 4 cVDPV oubreaks with a total of 21 polioviruses amongst. One of these poliovirus was a WPV and 20 were circulating Vaccine Derived Polioviruses (cVDPV) (figure 3).

The cVDPV outbreaks were respectively in 2001 (1 case), 2002 (4 cases), 2005 (4 cases) and 2014-2015 (1 cases). Among these cVDPV viruses, 9 were cVDPV2 and 11 cVDPV1 all in 2014. Three out of the four outbreaks were due to a cVDPV2 (2001, 2002, 2005). Most cVDPV outbreaks occured in Toliary region which registered 9 (43%) out of the 21 polioviruses detected in the country. This region was the only affected region in the 2001, 2002 and 2005 outbreaks. The second most affected region was Androy with 5 cases (25%) of poliovirus. Other affected regions included Analamanga (1 PVS, 1997), Anosy (1 case), Boeny (2 cases), Menabe (1 case), Soida (1 case), South West (1 case). At the district level, Tshiombe in Androy Region and Tolagnaro in Toliary region registered the highest number of CVDCV cases with 4 cases each. Figure... summarizes the geographical distribution of Polioviruses detected in Madagascar from 1997 to 2017.

The last cVDPV was in 2015 in Sakaraha health districts. This was a contact of an AFP case with onset dated on the 14^th^ August 2015. The last Wild poliovirus outbreak was in 1997 with a date of onset on the 20^th^ October 1997. This was a type 3 Poliovirus detected in a five years old unvaccinated child in Antananarivo district.

Polio compatible clusters were registered in three districts out of 114 during the 2014-2015 cVDPV outbreak. These were Fianarantsoa II, Sambava and Bekily. No other cluster has been reported in the country since 2015.

## Supplementary Immunization Activities and Quality Assurance

From 1997 to 2017, a total of 19 supplementary immunization activities were conducted in Madagascar among which 3 were local immunization days (LID’s) and 16 were national immunization days (N1Ds). These included either 14 reactive and 5 preventive supplementary immunization activities. In all, 16 SlAs rounds targeted children below 5 years and three rounds in 2015 (September, October and November) targeted children up to 15 years. Overall 80, 418, 817 children were vaccinated during these SIA’s corresponding to 160 837 634 polio doses administered. Oral polio vaccine was mostly used during SIA’s in Madagascar during this period. A total of 15 S1A used tOPV (before the switch) and 4 bOPV after the tOPV-bOPV switch in 25 April 2016. IPV vaccine has not been used in Madagascar for SIA’s (Table 3).

SlA quality was evaluated using independent monitoring since April 2015. It was used in 10 S1A. The proportion of in-house children missed varied from 4% in January 2015 to 8 % in January 2016. Similarly, the proportion of out-of-house children missed varied from 5% in August 2015 to 11% in January 2017. The main reasons for non-vaccination were absence (38-65%) of children when vaccination teams visit homes, house not visited by vaccination teams (9-27%) and vaccination refusal (4-39%). We observed a progressive increase in the proportion of refusals with highest levels of 35% and 39 % registered during polio SIAs in 2017.

SIA performance evaluation using lot quality assurance sampling (LQAS) methodology, which provides a quick and reliable immunization campaign assessment, was introduced in Madagascar in march 2017 with 20(17.5%) and 37(32.4%) implementing districts in March and December 2017 respectively out of 114 districts in the country. About 43 (75, 43 %) districts out of the 57 districts surveyed passed the 80% LQAS threshold.

Efforts aimed at improving SIAS quality were update of polio microplans in 2015 using a bottom up approach, introduction of vaccination team supervisors, daily monitoring of SIAs and use of IM & LQAS results for timely adjustment of strategies. In addition, multi-sectorial collaboration was strengthened by setting up SIAs coordination teams at each levels of the health system led by the administrative and municipal authorities. Other initiatives were established to help improve vaccination coverage in high-risk areas including deployment of WHO and Unicef technical assistance before and during SIAs, and collaboration with humanitarian NGO’s. However, challenges remain in security compromised areas where more innovative approaches should be developed to make sure all these areas are covered.

## Discussion

### Routine immunization

Both administrative immunization coverage and WHO/ UNICEF estimates show gradual increase of national immunization between 1991 to 2007. Discrepancies between the two estimates is observed from 2008 to 2017 where WHO/UNICEF coverage estimates with discrepancies above 10% are considered acceptable. The year 2008 was dominated by a socio-political crisis which affected the country and had negative impact on the health system. Improvement of national immunization coverage from 1991 to 2007 was mainly linked to national authorities’ leadership. During the transition period, government institutions were weak, and activities were directly implemented when possible by partners on the field.

During the transition period between 2008- 2009, government institutions were weak, health personnel demotivated due to low salaries. The health system was affected and partners could only implement activities directly whenever possible. Drop of national immunization coverage estimates from 2008-2017 can be explained by the weakness of the health system which has not yet recovered from the crisis. This discrepancy also highlights the concerns about data quality, since demographic data used by the health system result from the last national census conducted in 1993.

The percentage of Acute Flaccid Paralysis cases among children aged 6-59 months who received >=3 doses of OPV provide an indirect opportunity to estimate OPV coverage in target populations using Immunization card and parental recall. Improved of AFP cases immunization status from 55% to 85% who received three doses of OPV shows the important contribution of the 14 SIAs conducted in the country following the 2014-2015 cVDPV outbreak. The effect of SIA’s on children immunity in Madagascar is in line with research conducted by Rakoto Andrianarivelo and al. in 2001.

Efforts to address the poor routine immunization coverage include, organizing mother and child health weeks twice a year all over the country. This is an opportunity for children catch up, identification of 34 health districts were RED strategy in being implemented with GAVI support, gradual coverage of health facilities with solar fridges and strengthening logistic capacities of front-line health facilities for outreach activities. Remaining challenges include improving immunization coverage in regions, which have been poor performing like Melaky (78%), Sud-East (79.8%) and Boeni (81.1%) and to address data quality issue related to non-updated demographic census data. Melaky region is the most unsecured and hard to reach region of the country. Tailored strategies will be needed in this region to avoid occurrence of cVDPV outbreak in the coming years.

### Surveillance I WPV epidemiology and laboratory

Epidemiology of the 21 polioviruses registered in Madagascar from 1997-2017 shows that 95.4% of poliovirus were cVDPV viruses indicating immunization gaps in the population and mainly in Toliary and Androy regions which registered 63% of the cases. Continue strengthening of immunization and surveillance activities in these regions should remain among the priorities to maintain polio free status in Madagascar.

AFP surveillance system shows remarkable progress in AFP surveillance indicators in Madagascar and achievement of main polio surveillance indicators during the last ten years. However, most important progress followed the last cVDPV2 polio outbreak in 2014-15. During the management of this outbreak WHO and UNICEF mobilized both national and international consultants who contributed in the improvement of active case search in the health facilities and in the community. They also contributed to the training of national surveillance focal points at different levels of the health system.

Non detection of WPV and cVDPV by environmental surveillance confirms polio circulation interruption declared by the fifth polio outbreak Assessment (OBRA) in November 2015. Also, absence of type2 polio sabin virus in sewage samples indicates tOPV to bOPV switch success.

Despite the improvement of AFP surveillance system, the lowest sensitivity of the surveillance system is seen in Itasy (1.42), Aloatra Mangoro (2.48) and Bongolava (2.56) regions. Late detection of AFP cases in Melaky, Sud-Est and Androy regions with less than 80% stool adequacy can expose to silent circulation of poliovirus. Tailored strategies and continuous efforts will be necessary in these regions to maintain polio free status.

### Supplementary Immunization Activities (SIA)

SIAs conducted in Madagascar since 1997 used either tOPV or bOPV. The IPV has never been used during SIAs. However, quality of these SIA is still to be improve as showed by Independent monitoring and LQAS results. The percentage of children missed has been above 5% during all these SIAs. Lot quality assurance sampling (LQAS) methodology, which provides a quick and reliable immunization campaign assessment, was introduced in Madagascar in march 2017 and shows few districts accepted.

Efforts to improve quality of SIA included updating microplans in 2015, setting up permanent vaccination teams in markets and public places, appointment of vaccination teams’ supervisors to monitor vaccination team. However, SIA improvement should focus on identifying and immunizing previously unvaccinated children improvement on preparedness activities, Monitoring and quality supervision of preparedness and implementation activities including accountability of national authorities at different levels. Lot quality assurance sampling (LQAS) should be extended to most of the districts with support of local GPEI partners. The high proportion of during polio SIAs in 2017 could be explained by an unprecedented urban pulmonary faced that year by the country during which there was some cultural challenges related to corpses management leading to rumours and population hostility towards health interventions including Immunization. Non-vaccination reasons reveal operational gap in the organization of SIA including communication, microplannification, vaccination team supervision and accountability of staffs and community volunteers involved in polio immunization activities.

Non-vaccination reasons indicate operational gaps in the organization of SIAs including communication, micro-planification, vaccination team supervision and accountability of staffs and community volunteers involved in polio immunization activities which are remaining challenges to address.

### Acceptance of Madagascar’s polio free status documentation by the Africa Regional Certification Commission (ARCC) for poliomyelitis eradication

The ARCC is an independent and the only body to certify the African Region to have eradicated Wild poliovirus. A remarkable progress has been made toward interruption of the transmission of Wild Poliovirus (WPV) in Madagascar. The Polio eradication activities in Madagascar were supported by WHO furthermore in the country’s documentation process through technical mission, verification visits and advocacy activities to the highest authorities. The country has met the polio free status criteria. Following in-depth review and acceptance of its polio free documentation, the ARCC declared Madagascar polio-free at the ARCC meeting held from 18-22 June 2018 in Abuja, Nigeria. The certification achievement presented by the country can be summarized as follow: (i) the last WPV case was detected in Madagascar in 1997; (ii) For more than the past 3 consecutive years (2015, 2016, 2017), the country achieved the certification standard surveillance performance with a Non-Polio AFP rate above 1/100 000 children below 15 years and a stool adequacy rate above 80%; (iii) The administrative OVP3 coverage has been above 80% (with WHO/Unicef estimate 78% average; (iv) The country has a robust polio outbreak and preparedness plan. This plan is regularly updated and was adapted to respond to the cVDPV detected in the country in 2015; (v) Th phase 1 containment activities have been implemented in line with GAPIII; (vi) The 3 polio committees (NCC; NPEC and NTF) are established and functional with quarterly meetings minutes, annual report on polio eradication activities and polio committees work plan shared with WHO AFRO.

Following acceptance of the complete documentation of Madagascar in June 2018, the Commission recommended the country to sustain the achievements with high level of commitment to further strengthen immunization and surveillance systems including prioritization of surveillance sites until global certification and beyond. This study give opportunities for future study/research on monitoring trend of surveillance and immunization performances years after a country is declared polio free by the ARCC, analysing reasons why some regions are prone to recurrent cVDPV2 outbreaks and indeep analysis on Routine immunization data quality.

## Conclusion

Madagascar’s achievement of polio free status was possible because of high political commitment and GPEI partner’s support. However, Socio-political instability, weakness of the health system and existing security compromised areas remain critical program challenges to address.

Achievement is highly dependent on financial and technical support provided by GPEI partners. Sustaining these results required progressive ownership of surveillance and immunization system by national authorities and health system actors at different levels of the health system.

Continuous commitment of Government and GPEI partners to strengthening routine and supplementary immunization activities in all areas of the country is needed as well as efforts to ensure high quality surveillance.

## Figures and Tables

**Figure 2:**
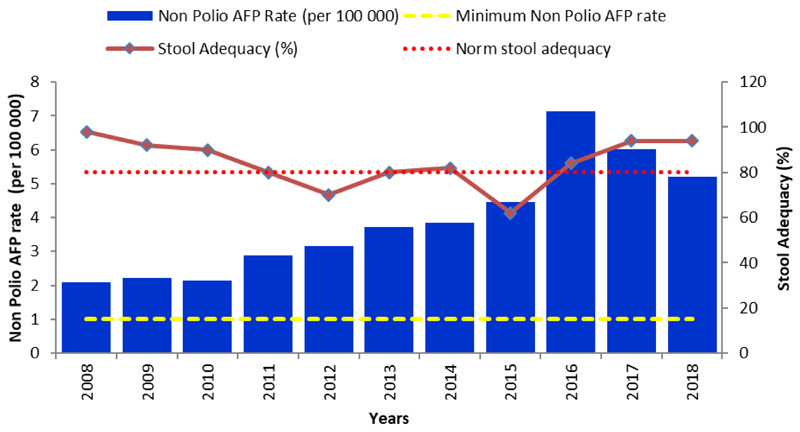
Non-Polio AFP rate and Stool adequacy in Madagascar from 2008 to 2018

**Table 1:** Polio vaccine administrative coverage (OPV3) trend by region in Madagascar, 2008-2017.

OPV 3 Administrative Coverage (%)											
Regions	2008	2009	2010	2011	2012	2013	2014	2015	2016	2017	Average (%)
ALAOTRA MANGORO	100	90	101	96	91	90	84	89	92	90	92,3
AMORON’I MANIA	87	87	96	90	85	86	86	77	87	86	86,7
ANALAMANGA	101	91	95	89	84	86	83	90	85	86	89
ANALANJIROFO	110	99	87	88	88	100	92	79	91	100	93,4
ANDROY	88	112	109	97	94	77	88	76	91	77	90,9
ANOSY	95	89	98	92	85	90	94	70	96	90	89,9
ATSINANANA	94	87	81	90	88	96	88	87	85	96	89,2
BETSIBOKA	96	94	74	96	91	82	100	103	92	82	91
BOENI	75	74	67	71	86	75	100	96	92	75	81,1
BONGOLAVA	90	100	92	96	83	91	75	76	73	91	86,7
DIANA	97	81	87	87	85	82	94	96	94	82	88,5
HAUTE-MATSIATRA	84	90	75	88	80	91	88	85	99	91	87,1
IHOROMBE	108	96	86	99	92	97	88	89	98	97	95
ITASY	89	99	90	91	86	80	78	130	88	80	91,1
MELAKY	83	109	55	71	76	81	76	62	86	81	78
MENABE	91	74	75	82	84	98	84	71	94	98	85,1
SAVA	77	89	77	88	81	89	91	85	87	89	85,3
SOFIA	83	92	76	80	99	84	93	82	96	84	86,9
SUD-EST	86	90	56	87	87	89	74	62	78	89	79,8
SUD-OUEST	75	71	80	79	81	86	101	81	85	86	82,5
VAKINANKARATRA	88	92	94	89	87	100	101	98	93	100	94,2
VATOVAVY	78	76	84	92	84	85	84	86	102	85	85,6
MADAGASCAR	90	89	83	88	86	89	89	86	90	89	87,9

**Table 2:** Key AFP Surveillance indicators trend at National level in Madagascar from 2008 to 2017

Year	Population (<15 years)	Number of expected AFP cases	Total Number of AFP notified	Number of confirmed Poliovirus	Total Number of Non Polio AFP	Non Polio AFP Rate	Number of adequate AFP cases	
							Nombre	%
2017	11.488.725	230	692	0	692	6,02	645	93,2
2016	11 012 186	220	788	0	785	7,13	669	85
2015	10 722 674	214	505	10cVDPV 1aVDPV	480	4,48	307	60
2014	10 874 000	217	415	1cVDPV	414	3,81	348	83,9
2013	10 577 820	212	392	0	392	3,71	318	81,1
2012	10 391 653	208	328	0	328	3,16	226	68,7
2011	10 109 792	202	291	0	291	2,88	234	80,4
2010	9 763 235	195	210	0	210	2,15	187	89
2009	9 497 239	190	210	0	210	2,21	193	91,9
2008	8 752 611	175	183	0	183	2,09	179	97,8

**Table 3:** Supplementary immunization activities (SIAs) conducted, and number of oral poliovirus vaccine used in Madagascar, from 1997.to.2017

Year	SIA type	Vaccine antigen type	Target Population	No. children vaccinated	Administrative coverage (%)	% children Missed by IM		Nber (%) of Districts achieving ≥90% coverage on LQAS* (%)
						House (%)	Outhouse (%)	
2017	NID	OPVb	4 140 359*	4 091 432	99	7	11	20 (15)
2017	NID	OPVb	4 616 093	4 637 339	100	6	8	37 (27.02)
2016	NID	OPVb	4 761 374	4 748 909	100	8	11	-
2016	NID	OPVb	4 761 374	4 909 958	103	8	11	-
2016	NID	OPVt	4 761 374	4 677 891	98	5	8	-
2015	NID	OPVt	11 018 838**	11 823 871	107	4	5	-
2015	NID	OPVt	11 018 838**	11 691 982	106	4	7	-
2015	NID	OPVt	11 018 838**	11 136 641	101	6	8	-
2015	NID	OPVt	4 284 823	4 295 027	100	6	7	-
2015	NID	OPVt	4 284 823	4 202 695	98	-	-	-
2014	sNID	OPVt	980 496	932 497	95	-	-	-
2005	sNID	OPVt	507 658	655 760	129.2	-	-	-
2005	sNID	OPVt	507 658	687 899	135.5	-	-	-
1999	NID	OPVt	2 668 232	3 259 806	122.2	-	-	-
1999	NID	OPVt	2 668 232	3 136 415	117.6	-	-	-
1998	NID	OPVt	2 590 518	2 852 126	110.1	-	-	-
1998	NID	OPVt	2 590 518	2 757 297	106.4	-	-	-
1997	NID	OPVt	2 515 067	2 809 846	111.7	-	-	-
1997	NID	OPVt	2 515 067	2 546 517	101.3	-	-	-

* Target population reduced due to exclusion of three health district facing pulmonary plague outbreak

** SIAs targeted children under 15 years.

## Summary

**What is already known about this topic?**

- The last WPV 1 in Madagascar was in 1997 and the country achieved polio free status in June 2018;
- Poliovirus transmission was interrupted in Madagascar in November 2015, following the conclusion of OBRA5
- 1PV Vaccine was introduced in Madagascar in 2015 and Switch to bivalent oral poliovirus vaccine completed on the 25th April 2016.

**What is added by this report?**

- Regions and districts most affected by Polio outbreaks from 1997 to 2017 in Madagascar are identified and should be closely monitored until global polio certification.
- The last cVPDV1 outbreak in Madagascar in 2014-15 was a critical step in the improvement of the quality of Polio eradication activities in the country.
- Madagascar polio history is marked by recurrence of cVDPV outbreaks than WPV outbreaks suggesting persistent immunity gaps and poor routine immunization. The risk of having other cVDPV outbreaks should be reduced by strengthening Routine Immunization programme with emphasis in poor performing areas and addressing data quality issues revealed by discrepancies between administrative coverages and WHO & Unicef estimates.
- Security compromised areas remain a critical challenge for national authorities and GPE1 partners in the country and need tailored strategies.

**What are the implications for public health practice?**

- Maintaining polio free status in Madagascar will require addressing persistent gaps and challenges in surveillance and ensure vaccinating every missed child during Routine Immunization services and S1As.
- Security compromised areas should be targeted by innovative strategies.
- Operational gaps remain on the organization of SIAS and contribute most on reasons of non-vaccination of children (House not visited, absence, refusals). Addressing these gaps will enable improvement in the quality of upcoming S1AS and population immunity
